# Traveling Memoranda from the Editor

**Published:** 1836

**Authors:** 


### III.—ORIGINAL.

### THE WESTERN JOURNAL.

*E Sylvis Nuncius.*

CINCINNATI, OCTOBER 1st, 1836.

### 1.—Traveling Memoranda—From the *Editor.*

*St. Louis, Mo., June* 6, 1836.—The great relative importance of St. Louis, among the new cities of the expanded and flourishing West, must render a passing *medical* notice of its physical and social condition, acceptable to the readers of the Western Journal. St. Louis is strictly the capital of the *Far West*. Situated beyond the original limits of the Union, on the further bank of the Mississippi, and at the edge of the great eastern declivity of the Chippewyan, absurdly called Stony Mountains, a traveller from the central part of the Valley of the Ohio, finds himself, on landing at this place, quite without the limits of his former associations, and feels many new and interesting emotions. The expanded, deep, and rapid river that washes the rocky *quay* of the town, and carries onward to the ocean the sandy and argillacious *debris* of the extensive diluvial formations, through which the Missouri rolls its waters, presents him with geological specimens of the great western *terra incognito*, and animates him to inquire into the composition of those turbid waters, and their probable influence on the health of those who drink them, and of those who live where the alluvion of the stream is accumulated. These deposites are constantly changing their places, so that families which were once on the margin of deep waters, are now in the neighborhood of extensive *bars*, composed of sand, clay and uprooted trees, confusedly blended, and exposed in July, August, September, and October, to the rays of the sun. Extensive deposites of this kind, annually extending in some directions and contracting in others, exist adjacent to the lower parts of the city, and must be looked upon as a source of autumnal disease.

Immediately opposite this bar, there enters the Mississippi a small stream, the bed of which for a quarter of a mile abounds in slime, which lying to the south of the city, must, to a certain extent, be regarded as injurious to health. A high mill dam thrown across this stream, forms, above and to the south-west of the city, a sinuous lake of deep clear water, surrounded by high, verdant banks which, therefore, cannot be supposed to exert any great degree of mischievous influence on the health of the city, although lying in the course of its summer winds.

On the opposite side of the river, is the American Bottom, extending from the mouth of the Kaskaskia river, to Alton, above the junction of the Missouri with the Mississippi; and having, opposite St. Louis, a width of six or eight miles. I crossed this *bottom* to the Illinois *bluffs*, and found it abounding in shallow lakes, lagunes and bayous, which, during the rains of spring, accumulate a great deal of water, much of which is lost by evaporation and infiltration in the course of the summer. This *bottom* is among the most fertile spots of the whole earth; and in a century, when a system of draining and tillage shall have been established in it, will become healthy, but, at present, it is infested with musquitoes, and intermittent fevers; the latter followed by enlarged viscera and dropsical infiltrations. As the Mississippi is two-thirds of a mile wide, and the Illinois alluvion lies directly to the east of St.

Louis, it cannot be regarded as contributing much to the unhealthiness of the place.

The city is built on a gentle declivity, highly favorable to cleanliness; but its municipal authorities do not seem to pay any special attention to the "art and mystery" of *scavengering*.

The substratum of its site and of the country in its rear, is a secondary siliceous lime stone, abounding in beds of bituminous coal. The elevation of the highest part of the city, above the mean height of the river, may be eighty or one hundred feet—that of the adjoining country, as much more. The surface of the latter is undulatory with basins, from which the rains pass into the cavernous lime stone below, to burst out in large perennial springs, which, however, are not very numerous. On the whole, the topography of St. Louis seems to me rather more favorable to health, than that, either of Cincinnati or Louisville. Six miles west of the city, there is a Sulphur Spring, which, however, has not yet become a place of fashionable resort. The fuel consumed by the citizens is a mixture of wood, and bituminous coal abounding in pyrites. At present the former predominates, but it is easy to foresee that at no distant time the latter must be chiefly used.

The city is supplied with water from the Mississippi, pumped by steam machinery into a basin constructed on one of the ancient mounds, which abound on either bank of the river. Taken from the Missouri side of the stream, it has, of course, the muddiness of that river, but by subsidence and filtration it is rendered transparent and pleasant.

The population of St. Louis, consists of creole French, quite a minority; negro slaves, not very numerous, *apparently* not so many, compared with the entire population, as the free negroes of Cincinnati; German and Irish emigrants, the former much the most numerous; and, lastly, emigrants from the various states, from New England to the Carolinas inclusive. In addition to all these, there are always sojourning in it, a great many of boatmen, and a number of hunters and furtraders. Thus, the elements of its population, are extremely diversified; and many fine opportunities will be created for studying the varieties of physiological character, which must necessarily result from an extensive commingling of the different *castes*.

The physicians of the city are, as in the other towns of the West, in good proportion to the number of the people. The majority of them appear to be "new comers," and gentlemen in early life, educated in various schools. They are not *entirely* without some of the intestine difficulties which perplex the faculty, in all new towns as well as old; but a laudable effort has lately been made, to obviate jealousies, and create a professional *esprit du corps*, by establishing a society, which is styled the MEDICAL SOCIETY OF MISSOURI. Its officers are *Dr. Farrar*, the oldest and most popular physician of the place, president; *Dr. H. Lane*, likewise an old and influential physician, vice president;—*Dr. Johnson*, corresponding secretary;—*Dr. Brown*, recording secretary; and *Dr. Bolling*, treasurer.

This society has the distinction of being the first ever established west of the Mississippi river. I had the advantage of being present at one of its meetings, and listened to a debate on the pathology and treatment of Epidemic Cholera, in which the different gentlemen who spoke, evinced the same diversity of opinion, that prevails in other and older medical communities. I should not infer from any thing which I then heard, or have learned since coming into this region, that there is reason to fear a return of the epidemic, the present summer. I trust indeed, that it has expired in this latitude, if not in the southern border of the United States, where however its cause may, perhaps, still linger.

St. Louis has one public infirmary, called the St. Louis Hospital. It is in the management of the SISTERS OF CHARITY, by whose exertions in a great degree, it was founded. It has attached to it, an entire square in the southern, but not the healthiest part of the city, presented for the purpose, by the late Mr. John Mullanphy. The edifice, erected by contributions from the citizens of St. Louis, is small, but well adapted to the object, and furnished and kept in a style of great comfort and neatness. Many of the sick paupers of the city are sent to it at the public expense. In addition, strangers who have the means of payment, are permitted to enter, and selecting their own physicians, secure to themselves the care and sympathy of the respectable ladies, who have dedicated their lives to this labor of charity. The medical attendants are Dr. Campbell and Dr. Call.

*Monte Sano*, *July* 15, 1836. The phrase "Mountain of Health," may, I presume, be taken as a fair translation of the two Latin words, which indicate the spot from which I now write. But where is Monte Sano? Why, inquisitive reader, just two miles east of the *ancient* and tranquil town of Huntsville in North Alabama; and its summit towers about 1000 feet above the table land on which said town is built. As the Cumberland mountain stretches down to the south west, it explodes, so to speak, into several masses, more or less disjointed, and Monte Sano is one of these. Its flat, sand stone summit, overshadowed by stately oaks and chesnuts, is bounded by mural precipices, from one or more of which, springs of pure water burst out with a temperature proper to the latitude of 39° instead of 35°.

Such is the summer retreat of the people of Huntsville, or of such of its wives and daughters, as prefer the labor of climbing into a higher elevation, to that of traveling into a higher latitude, or of remaining at home below.

Although of limited extent, the area of Monte Sano, is great enough to afford comfortable hot weather abodes, for several thousand persons; and not being at this time "in the way" of prescribing "drugs and medicines" I beg leave to send to such of our drooping friends in Louisiana, Mississippi and South Alabama, as may see the Western Journal, the following prescription:

℞. *A residence on Monte Sano, for the three summer months.*

This is especially adapted to all such as cannot make long voyages to the north; or have no more sagacity when they make them, than to continue in the deep valleys of the rivers which they ascend.

But Monte Sano is not the only salubrous mountain of this region. The various chains known under the names of Blue Ridge, Cumberland, Clinch, Walden, Racoon and Lookout, through which the beautiful Tennessee and its numerous tributaries meander, would afford summer residences for a nation. They are the back and upper country of the South, its north-land, a subalpine region, where the bilious and wilting planters of the great southern declivity, from the Saluda to the Arkansas, might congregate in hot weather, and enjoy the luxury of cool and fresh breezes — pure and cold water — mountain rambles — new companionships, and thunder storms under foot as well as over head.

Epidemic Cholera.—In traveling from Memphis on the Mississippi, in the Western District of Tennessee, through North Alabama, as far as Knoxville in East Tennessee, I have every where been assured, that the Epidemic Cholera has not yet visited the people. Memphis suffered, but not in any remarkable degree. Most of the towns in the interior of West Tennessee have not been scourged, even with the far famed, and much talked-about premonitory symptoms, in other words with *the* disease in a mild form. The same remark is applicable to North Alabama, on each side of the Tennessee river, embracing Florence, Tuscumbia, Courtland, Decatur, Triana, Athens and Huntsville; while many towns in Middle Tennessee and in the south western portions of Kentucky, were sorely afflicted. To what should this exemption be attributed? To the latitude? No, for the epidemic has appeared both north and south of this region. To the elevation above the level of the sea? No, for the bed of the Tennessee river is intermediate in this respect, between the beds of the upper Ohio and the lower Mississippi. To the composition of the soil? This question is not so easily answered; for throughout almost the whole region, from the Cumberland mountain to the Mississippi river, the soil is of the red color, which seems to indicate a vast quantity of the *peroxide* of iron, and is regarded as eminently favorable to the growth of cotton. Underneath this stratum the rocks, it is true, are in some tracts, as the Western District just mentioned, a conglomerate of free stone, while in other portions, as North Alabama, they are chiefly calcarious. Still it must be admitted, that the soil itself may have had an influence in warding off the pestilence. Has the water of this extensive region exerted an influence? None whatever; for part of it is from wells and part from springs—part is soft and part hard—freestone and limestone springs being scattered over the whole region. Should the exemption be ascribed to the absence of marsh or paludal tracts? By no means; for although the general aspect of the country is broken and the surface dry, still many of the smaller rivers, especially in the Western District, as well as the Tennessee river, have wide alluvial lands, which are annually inundated, and subject to the autumnal diseases, usually met with in such localities. Was it any thing in the modes of living of the people of this region that preserved them? Certainly not for they live, like their surrounding neighbors, on a most copious larder; and a corresponding supply of hot bread—the temperature of which is proportionate to the warm hospitality of the South. Is it the want of trade and commerce, and their great high ways? It is not; for steam boats ply on the Tennessee river from Paducah, at its mouth, to Knoxville in the mountains, a distance of 700 miles, and many great routes of travel and migration, intersect the country in various directions, while no *Cordon Sanitaire* has been thrown across them; no quarantine imposed; no non-intercourse established. To what then *can* we ascribe the exemption of this region? I hope some of its numerous inquiring and intelligent physicians, will make the discovery, and transmit it for publication in the Western Journal.

Feather Beds. It is bad enough to sleep on feathers in summer, when one lives far in the north; but to be delivered over to such a fate, in the latitude of 34°, is deplorable. Every where in this region, the taverns, and, in general, the houses of the people, are furnished with feather beds, for June,

July and August, not less than for January. The medical gentlemen of this country should raise their voices against this absurd and enervating custom. A hard bed for hot weather, referring either to health or comfort, should be the motto of the whole South.

Steamers.—Although this region has protected itself against the Cholera, it has been out-generaled by the steamers, who, like the Influenza of the year 1807, have advanced regularly from the north-east to the south-west. In every town, we as certainly see their signs, generally lettered with the words "*Botanical Doctor*," as on the Ohio or the Hudson. No Empyricism in modern times, has been so diffusive: happy would it be for society, if none were more permanent. Steam doctoring is already on the decline in this region, as well as in its native north.

Physicians.—It may not be uninteresting for me to say, that from Cincinnati to this spot, by way of St. Louis, I have met with physicians every where that are not graduates; and with not a few, who have never attended even one course of lectures. It is quite a mistake, that too many young men study medicine, unless they could be restrained from abandoning the profession. Of the graduates, where I have been, the plurality are *alumni* of the University of Pennsylvania, and next in rank is the Transylvania school; I had presumed the reverse to be the fact.

Compensation of Physicians.—The practitioners south of the Ohio river, receive much better fees than those to the north; but then the price of every thing in the former, is higher than in the latter. I believe, however, that the profession is on a better footing, more respected, more influential, and more accumulative of property in the valley of the Tennessee, than in that of the Muskingum, Miami, Wabash or Illinois. Nevertheless, even its ablest members are perpetually leaving it for more lucrative pursuits. For example, in Huntsville, there are two or three physicians who have withdrawn from the practice; and, even Dr. F—— who is generally and justly acknowledged to be at the head of the profession, in this Valley, speaks of devoting himself, although in the meridian of life, to some other pursuit. This premature renunciation of what men have laboriously qualified themselves to prosecute, is manifestly not without a cause; and society should inquire into its nature, and the means which may be employed to countervail it. The period of greatest usefulness, in the life of a physician, extends from 40 to 60—he is not a man of wisdom before the former, nor of energy, after the latter term. All who reach the one should be held on to the other, not on their own account, but that of the community. But to secure this adherence, their honors and emoluments must be made equal to what are gathered by other cultivated and laborious men. Human nature requires this, and will not be satisfied with less.

(To be continued.)

---

### 2.—The Cincinnati College.

### *Medical Faculty.*

During the present Summer and Autumn, the Faculty have greatly augmented the means of instruction in this department. An additional building has just been finished, in which are an apparatus room and working laboratory, for the professor of Chemistry, an apartment for the Museum of Anatomical preparations, the number of which, especially in pathological Anatomy, has been greatly increased; and a Dissecting Room of the largest class, which is furnished with every thing necessary to the successful prosecution of practical Anatomy.

They have, also, in connexion with several of the physicians of the city, founded a Library of ten or twelve hundred volumes, which will prove especially useful to students who may remain in Cincinnati during vacation.

Lastly, they have established a general Infirmary, under the title of THE CINCINNATI HOSPITAL. Which is intended as a school of clinical and surgical practice, for the pupils of the Institution.

DR. WILLARD PARKER, of New England, an experienced and eloquent teacher, who in the month of May was appointed professor of SURGERY, in this department, to supply the place of Dr. Jameson, will, as he has written, start for Cincinnati about the 20th of October, and of course be present at the opening of the session, on the last Monday of that month.

Students who intend to graduate, should recollect, that they must register their names with the Dean, by the 20th of November; for none will be considered as attending a full course who do not enter before that period, unless they were detained by causes that are satisfactory to the Faculty.

*Law Faculty.*

The Law School of the Cincinnati College, composed last year of the Hon. JOHN C. WRIGHT, late Supreme Judge of the State of Ohio, and of TIMOTHY WALKER and JOSEPH S. BENHAM, Esqrs., has lately been strengthened, by the addition of EDWARD D. MANSFIELD, Esq., author of the "Political Grammar of the United States." This very able Faculty will commence its session on the first Tuesday of November, by public introductory lectures in the chapel of the College.

*Academical Faculty.*

The Trustees of the College have recently revived this Faculty, by the appointment of the distinguished Professor MCGUFFY, of the the Miami University, to the PRESIDENCY, and the chair of Moral Sciences. The other chairs, embracing a comprehensive course of studies, adapted to the requirements of the West, have been filled with the following gentlemen—Rev. ASA DRURY, of the Granville Institution, Ohio; ORMSBY M. MITCHEL and EDWARD D. MANSFIELD of this city, and CHARLES DAVIES, of the United States Military Academy. The three former are regarded, where they are known, as eminently qualified for their respective stations; while the last, by his teachings and writings, has placed himself in the estimation of the whole country, among the most distinguished of its scientific professors. His acceptance has not yet been received.

The President and Faculty, will we are informed, be installed into office, in the latter part of October; and open their first session, at the same time with the other Faculties of the Institution. We shall only add, that Professors Gross, Rogers, and McDowell will give to the Senior Class, instruction in Physiology, Chemistry and Anatomy, as branches of general education.

---

### 3.—The Cincinnati Hospital.

In the preceding article we have *mentioned* the Cincinnati Hospital, but a more extended notice may, perhaps, be acceptable to our distant readers. It is designed to be a school of clinical practice—medical, surgical and obstetrical. Two of the professors will visit it daily (except on Sundays) from two to four o'clock in the afternoon, for the purpose of prescribing, operating and delivering clinical lectures. During these hours there are no lectures in the College; so that the same number that were delivered last year, will be given hereafter; while the advantages of hospital practice, on a new plan, will be experienced. In these institutions generally, throughout the United States, the custom is for students to make but two visits a week. But between public prescribing days so remote, many changes take place, and much is done which the students cannot witness. Of course, the instruction derived from each case, is far less, than if all its symptoms and treatment passed in review before them. By daily visits they will obviously derive, a far greater amount of advantage, than by a semi weekly attendance. To favor this frequent visiting, the Cincinnati Hospital has been opened in a large, well finished commodious house, directly across the street from the College edifice, in the very centre of the city.

The classes of patients which will be admitted are, 1. *Boatmen*, attached to steam boats, that pay to the surveyor of any western port from New Orleans to Pittsburg, the sum of 20 cents a month out of the wages of each *hand;* the Faculty having made arrangements to this effect with the government. This is a kind of embryotic national hospital. 2. *Strangers*, in indigent circumstances. 3. Persons of all kinds afflicted with maladies of the eye. The number of the latter class will undoubtedly be very considerable, as the patients of the Cincinnati Eye Infirmary will be turned over to this establishment. This public charity was chartered by the Legislature of Ohio, in the winter of 1827–8, but has been without an endowment. Notwithstanding this, a great number of patients, especially poor persons, have resorted to it for relief, and almost every operation, known to the ophthalmolsgists, has been performed in it with success. Hereafter *indigent* persons will not only receive medical and surgical aid, but be boarded, lodged and nursed, in the Cincinnati Hospital. 4. Still further, the Faculty have fitted up a prescribing room, for DISPENSARY patients of the city, who are unable to pay for professional attendance and medicines; but, when not so ill as to be confined to the house, may visit the establishment daily and receive both. Such will be the different classes of patients, who will be made the means of practical instruction to medical students. During the session of lectures, the prescribers will be the professors of surgery and the practice of physic—for the remainder of the year, the other professors, two at a time, will give attendance.

We may add, as general information, that an entire range of rooms, in the Hospital, are prepared for the reception of sick travelers, who may prefer the quiet and good nursing of such a situation, to the accommodations which are afforded in taverns.

### 4.—National Marine Hospitals in the West.

It was our intention to devote several pages of this number of the Journal, to a renewed consideration of this important subject: but other engagements have rendered it impracticable. We cannot, however, permit the number to go forth, without suggesting to our friends in the west and south, the great importance of a simultaneous effort upon the senators and representatives in Congress, from their respective states and districts. Considerable discussion was had in the committees of that body last winter, which has certainly prepared it for action, in the ensuing session. If nothing should, then, be accomplished, a new congress will have to take the subject up, and travel over the same ground with the last. Should the medical gentlemen of the country interested, bring their powerful influence to bear upon the members who represent them in the general government, an immediate and effective action might be the consequence. We should be happy if Dr. Campbell of St. Louis, the author of the project, would visit Washington the ensuing winter, and communicate personally with members on the subject. The profound attention he has given it, would enable him to do more for its final accomplishment, than any other individual; while the commercial rank of his own city, would seem, in an especial manner, to justify her in sending a qualified agent to give information, where it is so much needed.

We are of opinion that the existing marine regulations are by no means adapted to the interior; and that the continent and the ocean should each have its own system. At New Orleans, it is required as a preliminary to admission into the Seaman's Hospital, that the *boatmen* from the upper country, should have *protections!* Why do not Congress abolish this absurdity, by creating new methods adapted to the interior? There should, indeed, be the continental river fund, and the marine fund; and the privileges of boatmen should be uniform, throughout all the commercial towns of the lakes, the Mississippi, and the tributaries of both.

### 5.—Lectures on Mental Philosophy for Students of Medicine.

President McGuffey, whose successful devotion to the abstruse science of mind, is well known to all who have had an opportunity of listening to his *prelections*, will deliver a course of lectures on Mental Philosophy, to the students of the three departments of the Cincinnati College; which to the medical, from an arrangement made by the professors of that branch of the Faculty, will be gratuitous. We have often observed with regret, that many physicians are more ignorant of the philosophy of the mind, than of any thing else connected with the human constitution; and that many of them, have not even perceived the necessity of studying the laws of man's nobler part. All this is wrong. The reciprocal influence of the mind and body, is a fact which should never be forgotten by the physician; and, indeed, it is not; but how few have made it a study! How few have had a realizing sense of its importance! Every enlightened and experienced physician, has seen diseases produced by mental emotion, protracted by the same cause in spite of his physical remedies, or, finally removed by the rise of an opposite emotion. But it is in the study and treatment of the innumerable varieties of mental aberration, that the value of this branch of physiological science appears in its greatest magnitude; and no practitioner should ever approach the unfortunate subject of one of these alienations, without a familiar acquaintance with the principles of the philosophy of the mind as well as the body. Finally, there is still another and very different reason, for cultivating intellectual philosophy. Physicians are the teachers of medical students, and to teach successfully, they must be well acquainted with this branch of science.

